# A Systematic Review of the Effect of Neurofeedback in Cancer Patients

**DOI:** 10.1177/1534735419832361

**Published:** 2019-03-05

**Authors:** Madeleine Hetkamp, Jasmin Bender, Nadine Rheindorf, Axel Kowalski, Marion Lindner, Sarah Knispel, Mingo Beckmann, Sefik Tagay, Martin Teufel

**Affiliations:** 1 Clinic for Psychosomatic Medicine and Psychotherapy, LVR Hospital Essen, University of Duisburg-Essen, Essen, Germany; 2 Neurofit Academy for Therapy and Training, Krefeld, Germany; 3 Department of Dermatology, University Hospital Essen, University of Duisburg-Essen, Essen, Germany

**Keywords:** cancer patients, postcancer survivors, neurofeedback, EEG biofeedback, psycho-oncology

## Abstract

**Introduction:** Neurofeedback (NF) or electroencephalogram (EEG)-Biofeedback is a drug-free form of brain training to directly alter the underlying neural mechanisms of cognition and behavior. It is a technique that measures a subject’s EEG signal, processes it in real time, with the goal to enable a behavioral modification by modulating brain activity. The most common application of the NF technology is in epilepsies, migraine, attention-deficit/hyperactivity disorder, autism spectrum disorder, affective disorders, and psychotic disorders. Few studies have investigated the use of NF in context of psychosomatic illnesses. Little is known about the use in cancer patients or postcancer survivors despite the high number of this patient group. **Objectives:** We here provide a systematic review of the use and effect of NF on symptoms and burden in cancer patients and long-term cancer survivors. **Methods:** In conducting this systematic review, we followed the guidelines of the Preferred Reporting Items for Systematic Reviews and Meta-analyses (PRISMA) Statement. **Results:** Our search resulted in only 3 experimental studies, 1 observational study, and 2 case reports. Given the heterogeneity of the intervention systems and protocols, no meta-analysis was conducted. **Conclusion:** Altogether, there is initial evidence that NF is a complementary, drug-free, and noninvasive therapy that has the potential to ameliorate symptoms in this patient group, such as pain, fatigue, depression, and sleep. Further studies are highly needed.

## Introduction

According to Micoulaud-Franchi et al, the technique of electroencephalographic neurofeedback (EEG NF) emerged in the 1970s and it measures a subject’s EEG signal, processes it in real time, extracts a parameter of interest, and presents this information in visual or auditory form. The goal is to enable a behavioral modification by modulating brain activity using concept of “voluntary control.”^1(p. 423)^ Previous studies in the 1960s confirmed that alpha-blocking could indeed be conditioned^2,3^ and that EEG synchronization as well as behavior could be modulated through operant conditioning.^1^

Neurofeedback (NF) or EEG-biofeedback is a “noninvasive, drug-free form of brain training.”^4(p. 318)^ The most common application of the NF technology is in stroke, epilepsies, attention-deficit/hyperactivity disorder, migraine, chronic insomnia, autism spectrum disorder, major depressive disorder, anxiety disorders, addictive disorders, and psychotic disorders.^1^ Neurofeedback is an innovative complementary and alternative medicine (CAM) therapy that is scientifically based.^4^ It allows the brain to learn and relearn self-regulation skills,^5^ which have clinical relevance, because brain wave modulation leads to symptomatic changes.^4^ Biofeedback, by which NF is included, works as a psychophysiological intervention through the mediation of cognitive changes,^6^ such as the improvement of self-efficacy^7^ and coping skills. A recent systematic review^4^ summarized the NF training aimed to change the electroencephalographic amplitude by the principles of operant and classical conditioning. Othmer and Othmer observed that the brain often responded to the training much too quickly, for example, in rapid and unexpected state shifts and symptom relief, to reasonably associate it with an operant conditioning reaction. They assumed that the brain derived more information from the signal than induced from the operant conditioning alone. They concluded that the brain in its executive function uses all given information for better regulation.^8^ For NF interventions, there are diverse approaches and protocols that can be used.

Few studies have investigated the use of NF in the context of psychosomatic illnesses, for example, to alter the perception of pain. Gannon and Sternbach^9^ found increased alpha activity (8-12 Hz) after training in a headache patient case study related to decreased intensity and duration of the headaches. Studies examining the effects of NF on chronic pain conditions such as fibromyalgia,^10,11^ trigeminal neuralgia,^12^ and complex regional pain syndrome type 1^13^ reported reductions in pain intensity, fatigue, depression, and anxiety. Several reviews and studies reported alleviation of fatigue^4,14^ and cognition^4^ by biofeedback.

There are many trials of efficacy of EEG NF in the fields of neurology and psychiatry, but many studies have significant methodological weaknesses.^15^ According to Micoulaud-Franchi et al, the following criteria are mandatory: (1) “a study design with controlled, randomized, and open or blind protocol,” (2) “a primary endpoint related to the disorder treated and assessed with standardized measurement tools,” and (3) “an identifiable EEG neurophysiological target, under-pinned by pathophysiological relevance.”^1(p. 425)^

It is assumed that oscillatory activity of neural populations represents a major communication mechanism of the brain^16^ and is related to cognitive functions.^17^ Abnormal oscillatory activity has been associated with psychiatric and psychological disorders. According to Micoulaud-Franchi et al,^1^ neurophysiological targets can be generally classified into 3 categories according to 3 general mechanisms: (1) arousal,^18^ (2) emotional valence,^19^ and (3) sleep.^20^ The particular clinical context can be used to determine the neurophysiological target selected for use with NF. On this occasion, arousal level can be related to the EEG power in certain spectral bands.^18,21^ For example, Haenschel et al could show that an increase in central frontal beta band (13-30 Hz) can be related to an increase in arousal.^22^ An increase in central frontal theta (4-8 Hz) band is related to a decrease in arousal with subjective sleepiness^23^ and an increase in occipital alpha (8-12 Hz) can be related to a relaxed state.^24^ Emotional valence has been related to EEG power asymmetry in frontal lobes.^25^ An increase in alpha power in the left compared to the right frontal cortex has been related to susceptibility toward negative emotions and is suggested as marker of risk for major depressive disorder.^26^ In general terms, anxiety disorder is related to an increase in arousal.^27^ Emotional valence targets are relevant for major depressive disorder, which is characterized by emotional negative bias.^28^

According to Luctkar-Flude and Groll,^4^ NF also has “the potential to alleviate multiple long-term symptoms reported by cancer survivors and improve their quality of life.”^4(p. 319)^ Global Cancer Incidence, Mortality and Prevalence (GLOBOCAN) 2012 reported there were 14.1 million new cancer cases, 8.2 million cancer deaths, and 32.6 million cancer survivors (within 5 years of diagnosis) in 2012 worldwide.^29^ In the United States, cancer death rates have declined 20% from their peak in 1991 (215.1 per 100 000 population) to 2009 (173.1 per 100 000 population).^30^ This decline in cancer mortality has contributed to an increased life expectancy. The numbers of cancer survivors and long-term cancer survivors have increased even more than those of new cases in well-developed regions. For Europe only, there were an estimated 3.45 million new cases of cancer and 1.75 million cancer deaths in 2012.^31^ As a consequence, there are probably approximately 32 million people worldwide (2012) who have had cancer in their lives.^32^ According to de Moor et al,^33^ approximately 13.7 million cancer survivors were living in the United States. Sixty-four percent of this population have survived 5 years or more; 40% have survived 10 years or more; and 15% have survived 20 years or more after diagnosis. The authors suggest that, over the next decade, the number of people who have lived 5 years or more after cancer diagnosis will increase approximately 37%.^33^

However, the usage of NF as integrative (psycho-oncological) treatment in cancer or postcancer therapy is rare. Luctkar-Flude and Groll describe that common physical and psychological symptoms experienced by cancer patients and cancer survivors are fatigue, cognitive impairment, pain, anxiety, and depression. The review investigation by Luctkar-Flude and Groll on fatigue and/or cognitive impairment shows the retrieval of only one single completed study, which investigated effectiveness of NF in the field of cancer survivors.^4^ This demonstrates that the oncology field has not yet evaluated this therapy. This review addressed nonspecific cancer-related symptoms but general fatigue and cognition. However, it has been shown that NF is effective in these symptoms. A transfer into practice is missing so far. Though NF has the potential to improve symptoms in cancer patients and cancer survivors, there is still a lack of NF investigations in this patient group.

Cancer patients suffer symptoms like pain, fatigue, cognitive impairment, anxiety, and depression.^4^ Falquez et al could show in a brain imaging investigation with brain tumor patients compared to healthy controls addressing emotional reappraisal of arousal and valence that several cerebral regions (right superior frontal gyrus, right dorsolateral prefrontal cortex, and the anterior cingulate cortex) were associated with cancer patient’s impaired downregulation of arousal.^34^ The ability to reappraise the emotional impact of events is related to long-term mental health. According to the authors, the development of evidence-based NF training would be of prime importance in patients suffering from depression, emotional dysregulation, and other types of postcancer impairments.^34^ Prinsloo et al also see NF as a noninvasive tool in the treatment of cancer patients. The authors argued that in cancer patients, pain may be caused by numerous reasons such as tumor progression, invasive treatments, infections, and generalized fatigue. These include various inflammatory, neuropathic, ischemic, as well as compression mechanisms. In addition, cancer pain may be an important predictor of survival, which can enable the progression of metastatic disease. They conclude that NF is a potentially effective, noninvasive, and economical tool in order to manage cancer impairments through modulating neural pathways.^35^

The purpose of this review will be to explore the effect of NF on various impairments and long-term symptoms commonly experienced by cancer survivors. So far, there is no such review that deals with various cancer impairments. The present systematic review seeks to summarize electroencephalographic NF evidence in cancer and cancer survivors. We will address the following main research questions:

1. Is EEG NF an intervention to alleviate cancer and postcancer impairments?
2. Does EEG NF modify cancer-related symptoms by modulating brain activity in cancer or postcancer patients?
3. Finally, which NF systems, approaches, and/or protocols have demonstrated effectiveness for management of cancer or postcancer impairments?

## Method

Data collection, reporting, and discussion were conducted according to the Preferred Reporting Items for Systematic Reviews and Meta-analyses (PRISMA) statement.^36,37^

### Information Sources and Search

In conducting this systematic review, we followed the guidelines of the PRISMA statement.^36,37^ The research team had experience in review methodologies. Inclusion criteria were defined a priori. A comprehensive systematic search of 4 databases was conducted: PubMed, PsycINFO, PSYNDEX, and EBSCOhost. The search terms used were neurofeedback or biofeedback or EEG biofeedback, and cancer or cancer patients or post-cancer patients or cancer survivor or cancer impairments or tumor.

We additionally performed a hand search through potentially relevant articles cross-cited in search results and inspected the reference list of a recently published review in the field of NF therapy.^4^ A study was eligible for inclusion in this review if it (1) reported on cancer patients or cancer survivors or patients with malignant tumors; (2) reported results of randomized controlled trials, nonrandomized controlled trials, controlled before and after studies, cohort, case control, or descriptive studies that assessed effectiveness of EEG biofeedback or NF therapy; and (3) was written in English. We excluded editorials, study protocols, reviews, expert opinion papers, and studies published as abstracts only.

**Figure 1.**
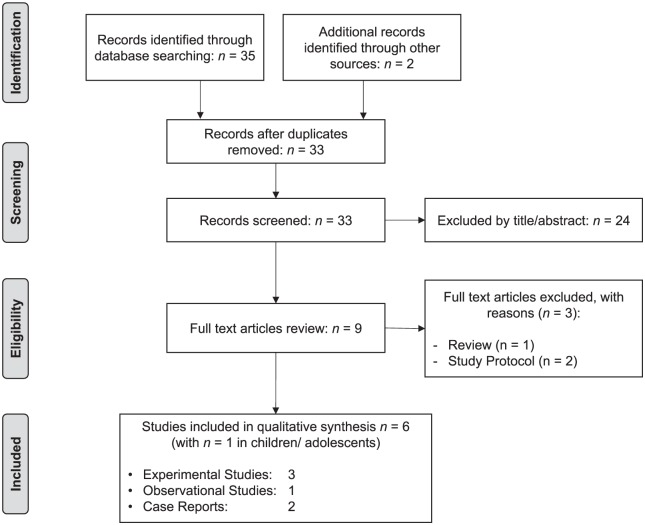
PRISMA search decision flow chart.

This article’s first and second authors independently assessed study eligibility following standardized eligibility criteria (see flowchart in Figure 1). The first author screened all search results by scanning article titles and abstracts. Afterwards, the 2 authors assessed and judged the full texts of potentially relevant studies with respect to eligibility. Interrater reliability was very good with κ = .97. Duplicate publications or overlapping reports were excluded from further analysis. The primary reviewer extracted data from the 6 included studies using an a priori developed data extraction form. Extracted data were transferred to synopsis tables to synthesize relevant data. The results were presented in separate tables by study design, experimental versus observational studies and case reports. The extracted data included the following specific details of significance to the review questions and objectives: reference, study design, patient population/sample, NF intervention (systems, protocols, number of sessions), outcome measures, results, conclusions, and limitations. Given the heterogeneity of the intervention systems and protocols, no meta-analysis was conducted.

## Results

### Findings

Our search resulted in 37 eligible records. We eliminated 4 duplicate records and excluded 24 records from further analysis during the screening process. From the remaining 9 studies, we excluded 3 studies, because they did not fulfil eligibility criteria (see Figure 1). Finally, we included 6 studies in this systematic review. Distribution of NF studies examining cancer patients is also presented in Figure 1.

Three experimental studies (see Table 1), 1 observational study (see Table 2), and 2 case reports (see Table 3) were synthesized in this review.

**Table 1.** Data Extraction Experimental Studies.

No.	Author/Date/Country	Study Design	Patient Population/Sample	Neurofeedback Intervention	Outcome Measures	Results	Conclusions	Limitations
1	Prinsloo et al (2017),^40^ USA	RCT with wait-list control period (after 4 months)	n = 71 cancer survivors with CIPN (87% female) n = 30 exp. n = 32 WLC 41-80 years	• EEGer software (EEG Education and Research) based on a qEEG • Sensor placement individual for each participant by comparative analysis and associated with report of symptoms • LORETA • minimum 2×/week • 20 sessions	Self-reports: • Brief Pain Inventory (BPI) • Pain Quality Assessment Scale (PQAS) • Clinically important change EEG neuroimaging for sensor placement	• The NF group demonstrated greater improvement than the controls on the BPI worst-pain item (primary outcome).	• NF appears to result in long-term reduction in CIPN symptoms.	• Diverse cancers epidemiology (73% breast cancer) • No cancer-related symptoms • Lack of a placebo control group • No analysis of current use of medications • Not easy to replicate due to individual sensor placement
2	Prinsloo et al (2018),^41^ USA	RCT with wait-list control period with 1-month and 4-month follow-up	n = 71 cancer survivors with CIPN (87% female) n = 30 exp. n = 32 WLC 41-80 years	• EEGer software based on a qEEG • Sensor placement individual for each participant by comparative analysis and associated with report of symptoms • LORETA • minimum 2×/week • 20 sessions	Self-reports: • Brief Pain Inventory (BPI) • Pain Quality Assessment Scale (PQAS) • Clinically important change • Cancer-related symptoms using the MD Anderson Symptom Inventory (MDASI) • Medical Outcomes Study 36-Item Short Form Survey (SF-36) • Brief Fatigue Inventory (BFI) • Pittsburgh Sleep Quality Index (PSQI) EEG neuroimaging for sensor placement	• Linear mixed model analysis revealed significant group × time interaction for pain severity. • A general linear model determined that the NF group had greater improvements in worst pain (primary outcome) and other symptoms such as numbness, cancer-related symptom severity, symptom interference, physical functioning, general health, and fatigue compared to the WLC group at the end of treatment and after 4 months (all *P* < .05). Effect sizes were moderate or large for most measures.	• NF appears to result in long-term reduction in multiple CIPN symptoms and improved post-chemotherapy QOL, fatigue, and symptom interference.	• Fluctuating sample size; at the 1-month time point, only 60% of the NF group and 41% of the WLC group completed assessments; at 4-month time point, 77% of the NF group and 88% of the WLC group completed assessments • Diverse cancers epidemiology (77% breast cancer) • Lack of a placebo control group • No analysis of current use of medications • Not easy to replicate due to individual sensor placement
3	de Ruiter et al (2016),^43^ the Netherlands	Double-blind randomized placebo-controlled trial with 6-months follow-up	n = 82 PBTS with neurocognitive complaints, >2 years posttreatment n = 34 exp. n = 37 PC 8-18 years	• Portable Brainquiry NF with BioExplorer software • At home or school • Sensor: Cz • Selection of most favorable training (Beta 1 up/SMR up/Sindles down) based on qEEG • 2×/week 30 sessions	Neuropsychological assessment Self-/parent- and/or teacher-reports: • KIDSCREEN-27 quality of life measure for children and adolescents • Dutch version of the Strengths and Difficulties Questionnaire (SDQ) for social-emotional functioning • Manual of the Self Perception (CBSK, CBSA) • Behavior rating inventory of executive function • Fatigue, attention in daily life. and sleep disturbance	• The results of this trial reveal no positive effects for NF over placebo feedback on any of the primary and secondary outcomes. • In both groups, PBTS improved over time on the majority of primary and some secondary outcome measures, with small to medium effects.	• The results indicated no specific treatment-effects of NF on neurocognitive functioning of PBTS.	• Patients were selected based on parent-reported complaints, not based on objective measures • The results of the objective neurocognitive tests suggest that participants may have been at greater risk for neurocognitive late effects than nonparticipants • Lack of transfer sessions to enhance generalization from the training setting into daily life • Sessions at home or school

Abbreviations: NF, neurofeedback; RCT, randomized controlled trial; CIPN, chemotherapy-induced peripheral neuropathy; exp., experimental group; WLC, wait-list control group; PC, placebo control; EEG, electroencephalogram; qEEG, quantitative electroencephalogram; LORETA, low-resolution electromagnetic tomography; PBTS, pediatric brain tumor survivors; SMR, sensorimotor rhythm; QOL, quality of life.

**Table 2.** Data Extraction Observational Study.

No.	Author/Date/Country	Study Design	Patient Population/Sample	Neurofeedback Intervention	Outcome Measures	Results	Conclusions	Limitations
1	Alvarez et al (2013),^38^ USA	Pre-post with wait-list control period	Breast cancer survivors n = 23 43-70 years	• NeurOptimal Nonlinear dynamic (NDL) approach • Sensors: C3,C4 • 2×/week • 20 sessions	Self-reports: • Functional Assessment of Cancer Therapy (FACT-Cog) • Functional Assessment of Chronic Illness Therapy–Fatigue (FACIT-Fatigue) • Pittsburgh Sleep Quality Index (PSQI) • Brief Symptom Inventory (BSI-18)	• Initially the sample showed serious dysfunction on all measures compared to population norms. • At posttest, the sample no longer differed significantly from the normative populations on 3 of 4 FACT-Cog measures and also the FACIT-Fatigue, and the psychological scales as well as on 3 of 8 sleep scales. • Repeated-measures ANOVAs revealed strongly significant improvements (*P* < .001) on all 4 cognitive measures and the fatigue scale.	• Breast cancer survivors showed significant baseline impairments in self-reported cognitive function and fatigue as compared with a normal population. • After 20 sessions of NF, their performance had improved to levels indistinguishable from population norms. • There were no dropouts from the study.	• Sample was small, exclusively Caucasian, and female • Serial neuropsychological testing and functional neuroimaging were not performed

Abbreviations: NF, neurofeedback; ANOVA, analysis of variance; EEG, electroencephalogram; qEEG, quantitative electroencephalogram.

**Table 3.** Data Extraction Case Reports.

No.	Author/Date/Country	Study Design	Patient Population/Sample	Neurofeedback Intervention	Outcome Measures	Results	Conclusions	Limitations
1	Nelson et al (2016),^39^ USA	Case report	A breast cancer survivor (female) 45 years	• Flexyx Neurotherapy System (FNS), involves minutely pulsed electromagnetic stimulation (max 30 Hz) • 19 scalp electrode sites • 1×/week • 10 sessions • 11th session 1 month afterwards	Self-reports: • Revised Piper Fatigue Scale (RPFS) • Center for Epidemiologic Studies–Depression Scale (CES-D) • Individual Symptom and Overall Activity Rating Scales	• Alleviation of fatigue and other potentially interrelated symptoms (cognitive clouding, sleep disturbance, pain, and negative mood/emotions) and overall greater activity level was sustained at 6-month follow-up.	• The woman showed alleviation of fatigue and symptoms like cognitive clouding, sleep disturbance, pain, and negative mood, and overall greater activity level.	• Serial neuropsychological testing and functional neuroimaging were not performed • No standardized self-reported measure of symptoms or cancer impairments
2	Prinsloo et al (2014),^42^ USA	Case report	A breast cancer survivor with CIPN (female) 60 years	• EEGer software (EEG Education and Research) based on a qEEG • Sensor placement by comparative analysis and associated with report of symptoms (initial P3, P4; 9th session: F8, F4; final protocol: T3, T4) • LORETA • min. 2×/week • 20 sessions	Self-reports: • Neuropathic symptoms on a numeric rating scale (NRS) Pre and post EEG neuroimaging (qEEG)	• Alleviation of neuropathy was reported since NF. • The training resulted in a transient visual improvement. • From baseline to post-20 session training, the authors were able to achieve an increase in alpha, which they hypothesized contributed to the client’s reported improvement of CIPN.	• CIPN seems to respond well to NF; however, visual changes secondary to chemotherapy are seemingly not as responsive as neuropathic symptoms in other regions of the body. • This case suggests that alpha increase after NF training did in fact correlate with improvement in visual symptom report.	• Serial neuropsychological testing • No standardized self-reported of symptoms or cancer impairments • Not easy to replicate due to individual sensor placement

Abbreviations: NF, neurofeedback; CIPN, chemotherapy-induced peripheral neuropathy; EEG, electroencephalogram; qEEG, quantitative electroencephalogram; LORETA, low-resolution electromagnetic tomography.

### Characteristics of the Included Studies

The 6 included studies were published in English between 2013 and 2018 in 2 countries. Five of the studies were conducted in the United States^38-42^ and one in the Netherlands.^43^ The study by Prinsloo et al (2018) represents the follow-up analysis of the study published in 2017.^40^ All experimental studies were randomized controlled trials (RCTs).^40,41,43^ The observational studies consisted of 1 pre-post study^38^ and 2 case reports.^39,42^

Three studies reported on results of patients with breast cancer,^38,39,42^ 2 studies on populations with heterogeneous diverse cancer epidemiology, but more than 71% breast cancer survivors.^40,41^ One study was conducted with pediatric brain tumor survivors.^43^ Sample sizes ranged from 71 to 82 for the experimental studies and from 1 to 23 for the observational studies. Participant ages ranged from 8 to 80 years. There was heterogeneity of primary outcome, for example, pain, quality of life (QOL), fatigue, and cognitive impairments.

### Neurofeedback Interventions Used in the Included Studies

A variety of NF systems and technologies were used in the included studies. The investigators of the University of Texas MD Anderson Cancer Center used a 19-electrode EEG cap with individual sensor placement for training by comparative analysis and associated with report of symptoms. They argued that treating one brain region with NF should lead to changes in other brain regions due to the large amount of connectivity between brain regions. Albeit not of the same magnitude of the change in the target region, NF would lead to improvements in areas such as general health, fatigue, and sleep, even if these regions were not directly targeted.^41^ They also expected a greater effect of NF by targeting different regions that may be more directly associated with symptoms such as fatigue or sleep disturbances.^41^ Two of the included studies used better replicable protocols with fixed sensor placements without qEEG (Flexyx Neurotherapy System and NeurOptimal Nonlinear dynamic approach).^38,39^ One case report employed a system that involves a NF intervention supplemented by minutely pulsed electromagnetic stimulation.^39^ Interventions ranged from 10 to 100 NF sessions 1 to 2 times per week, with most studies reporting 20 sessions twice per week.

### Effect on Cancer-Related Pain/Symptoms/Impairments

All the included studies except the pediatric record reported positive results for cancer-related symptoms, such as pain, QOL, fatigue, and cognitive impairments, measured by a variety of self-rating questionnaires. In addition, one study, a case report in a breast cancer survivor, achieved an increase in the quantitative electroencephalogram (qEEG) alpha bandwidth associated with improvement of symptom report.

In the pilot study and analysis by Prinsloo et al (2017) and Prinsloo (2018),^40,41^ applying NF protocols resulted in improvement in pain measured by the worst-pain item of the Brief Pain Inventory (BPI) as the primary outcome. All of the participants starting the NF program completed it. A linear mixed model analysis revealed significant group × time interaction for pain severity. The NF group benefited significantly over time, at the end of the treatment and even after 1 month, compared to control. Furthermore, a general linear model determined that the NF group had greater improvements in reducing worst pain and other symptoms such as numbness, cancer-related symptom severity, symptom interference, physical functioning, general health, and fatigue compared to the wait-list control group at the end of treatment and after 4 months (all *P* < .05). The authors found moderate or large effect sizes for most measures. Neurofeedback appears to result in long-term reduction in multiple chemotherapy-induced peripheral neuropathy (CIPN) symptoms and improved post-chemotherapy QOL and fatigue.

The single pediatric double-blind randomized placebo-controlled trial^43^ showed no effectiveness of the NF intervention used on neurocognitive functioning in pediatric brain tumor survivors (PBTS). The investigators used a portable NF system at home or school as well as selection of most favorable training based on qEEG and had a neuropsychological assessment. Patients were selected based on parent-reported complaints. The results of this trial reveal no positive effects for NF over placebo neurofeedback (the treatment modules were not based on the desired brain waves from the patients but on random generated signals) on any of the primary and secondary outcomes. Similar improvements were found over time for the 2 treatment groups, NF training and placebo training, on the primary neurocognitive outcomes (all *P* > .15). In both groups, PBTS improved over time on the majority of primary and some secondary outcome measures (eg, QOL), with small to medium effects. These results indicated no specific treatment-effects of NF on neurocognitive functioning in this trial.

A nonlinear dynamical training was reported in a pre-post study with a wait-list control by Alvarez et al.^38^ The authors demonstrated significant improvements in cognition and fatigue in a sample of breast cancer survivors experiencing serious cognitive impairment and fatigue (self-reports) following cancer treatment. At posttest, the sample no longer differed significantly from the normative population on 3 of 4 cognitive measures on the Functional Assessment of Cancer Therapy–Cognitive (FACT-Cog) and the Functional Assessment of Chronic Illness Therapy–Fatigue (FACIT-Fatigue) scales. Subjects demonstrated significant improvements on all cognitive measures (perceived cognitive impairment, comments from others, perceived cognitive abilities, and impact on QOL), the fatigue scale and the psychological scales (somatization, depression, anxiety, and global severity index), as well as on 6 of 8 sleep scales. According to the authors, improvements were generally linear across the course of training with 20 sessions and were maintained at the follow-up testing.

The NF protocol in Nelson and Esty’s^39^ case report in a 45-year-old female breast cancer survivor resulted in alleviation of cancer-related fatigue and symptoms like cognitive clouding, sleep disturbance, pain, and negative mood, and an overall greater activity level was sustained at 6-month follow-up. The investigators used a variant of electroencephalograph NF that involves minutely pulsed electromagnetic (EM) stimulation of brain wave functioning, which was administered in 10 weekly sessions.

The other case report in a 60-year-old female breast cancer survivor was submitted by the authors (Prinsloo et al^42^). Alleviation of chemotherapy-induced peripheral neuropathy was reported since NF. The training resulted in a transient visual improvement. The results indicated that chemotherapy-induced peripheral neuropathy seems to respond to NF; however, visual changes secondary to chemotherapy are seemingly not as responsive as neuropathic symptoms in other regions of the body. Furthermore, from baseline to post-20 session training, the authors were able to achieve an increase in alpha, which they hypothesized contributed to the client’s reported improvement of CIPN. This case study suggests that alpha increase after NF training could correlate with improvement in visual symptom report.

## Discussion

### Summary and Interpretation of Results

This is the first systematic review assessing the effects of NF on various impairments and long-term symptoms that are common in cancer patients.

The aim of this review was (1) to investigate whether EEG NF is an intervention that can alleviate cancer and postcancer impairments, (2) to describe the effect of NF modifying cancer-related symptoms by modulating brain activity in cancer or postcancer patients, and (3) to describe NF systems, approaches, and/or protocols that have demonstrated effectiveness for management of cancer or postcancer impairments.

Despite several limitations, we can conclude that all the included studies, except one record in pediatric cancer survivors, reported positive results of NF therapy for cancer-related symptoms. In addition, a case report achieved a modulation of brain activity by an increase in qEEG alpha bandwidth associated with improvement of symptom report. Regarding the third aim of this review, we found various inconsistent NF systems, approaches, and protocols. Interestingly, all included studies are reported in the last 5 years.

The most common use of NF training as a noninvasive, drug-free therapy is in the neurology, psychology, and psychiatric fields. Evidence of the effectiveness of this technique is available in many areas, for example, major depressive disorder and anxiety disorders. NF has clinical relevance in various explored fields. As a scientifically based complementary and alternative medicine therapy based on classical and operant conditioning, we should use these results and extend them to other areas. NF allows the brain to learn self-regulation skills and has influence on self-efficacy. Windthorst et al conclude that NF is easily and efficiently feasible within a circumscribed number of sessions. Patients report high acceptance and the procedure is very symptom-specific and easy to learn.^44^ According to the authors, biofeedback therapy provides a motivating approach especially for predominantly somatic or body-oriented patients. Self-effective behavior and treatment successes are quickly visible and can increase the commitment and the adhesion for the general cancer treatment. Furthermore, the increasing development and availability of portable biofeedback devices significantly improve the applicability and generalization capabilities beyond therapeutic institutions. The results of this review demonstrate some evidence that EEG NF has the potential to ameliorate symptoms in cancer patients and cancer survivors, even if data are very rare in this area. So, we can confirm the first research question of this review. Due to the increase of the absolute number of new cases of cancer during the last decades, as well as the numbers of cancer survivors and long-term cancer survivors, there would be a benefit of a drug-free intervention with minimal side effects. Cancer patients experience various symptoms as long-term and late effects of the cancer and its treatment, especially chemotherapy. This review provides evidence that symptoms like pain, fatigue, low QOL, cognitive impairments, sleep disturbances, and psychological strain can be modulated by NF therapy in cancer patients. However, the potential has not yet been exhausted in this patient cohort. It is important to translate the findings from other areas, such as neurology and psychiatry, into research and practice with symptoms of cancer patients and survivors. The number of cancer patients is high, so this technique should be used to a greater extent.

The next step would be to apply NF to different tumor entities. Most of the reviewed studies included breast cancer patients. The only one which did not was the pediatric study. The results are difficult to compare because of the selection of outcome measures and samples. Study designs vary widely and we have found several methodological weaknesses. So far, few RCTs have been published. Beyond the 3 experimental records, we found one study protocol in our research, in which analyses are still pending.^45^ The authors investigate postoperative pain reduction in lung cancer patients in a randomized controlled trial design, intervention versus usual care, and they use for example the international EORTC QLQ-C30 for quality of life. Our review also makes clear that there is little international research so far and there is little unity in the use of self-report instruments. In the reported studies, various inconsistent questionnaires and outcome parameters were used. Common symptoms, such as pain and fatigue, appear to be alleviated by NF, although not uniformly or standardized measured.

Cancer patients typically suffer symptoms like pain, fatigue, cognitive impairment, anxiety, and depression. This review generally indicates that these symptoms can be alleviated despite inconsistent measurements and methodological weaknesses. NF builds a drug-free task with few side effects to alleviate common impairments in this patient group. It is important to note that in clinical practice many cancer patients, especially patients with CIPN, cannot exert any physical activity to improve their symptoms. Consequently, NF could be a resource-saving alternative therapy.

Only one case report analyzed the pre and post qEEG data as primary outcome measure for associations or intervention effects potentially associated with the NF-intervention and reported an increase of the alpha bandwidth. The authors were able to achieve an increase in alpha, which they hypothesized is associated with the improvement. It is known that anxiety disorders are related to an increase of arousal and emotional valence targets are relevant for depressive disorder. It can be assumed that changes in cancer-related depression and anxiety involve similar changes in qEEG. Pre/post qEEG changes are a valid and reliable measure and would contribute to standardization. To delineate the mechanisms behind the effect of NF in cancer or postcancer patients, more research is needed.

Unfortunately, no comparisons to other drug-free interventions were found, though, for example, mindfulness-based stress reduction programs have been applied in the treatment of a number of conditions, including cancer. Besides the reduction of cancer symptoms, such as fatigue or pain, mindfulness exercises also effect an increase of the alpha band. According to several authors, NF trials should also be compared with other approaches that promote healthy brain self-regulation, such as physical activity and mindfulness-based stress reduction.^4,46^

Furthermore, we could not find uniform NF systems, approaches, and protocols that have demonstrated effectiveness for management of cancer or postcancer impairments. One reason is certainly the rare use of this therapy in this field. Despite these limitations, the results of the studies in this review show the potential of NF as complementary and alternative medicine therapy in cancer patients and survivors.

### Limitations

A limitation of this review is the small number of records. We found only 6 records. Methodological quality of the majority of the studies was only moderate, with only one blinded RCT included. Two records were based on the same study (one follow-up of the same cohort); 2 records were case reports. Confounding factors such as concurrent medication use or chemotherapies often were not described. Use of different outcome measurement tools as well as use of few standardized, international questionnaires and a lack of qEEG (pre/post) as outcome measure limit the ability to make comparisons between different NF protocols and approaches. Besides, the diversity of NF approaches and systems makes it difficult to categorize and explain the overall positive results.

## Conclusion and Future Directions for Research and Therapy

Despite several limitations, the overall positive findings suggest that NF as innovative CAM therapy could be helpful in alleviating cancer-related symptoms like pain, fatigue, and cognitive impairments. In other areas as neurology, psychology, and psychiatry, there is much evidence that EEG NF has the potential to ameliorate these symptoms. These promising results support the need for further research in this patient population. Currently, there is insufficient evidence that NF is an effective therapy for management of emotional distress, anxiety, and depression in cancer survivors. There is need to conduct studies with pre- and post-qEEG data as standardized outcome measurement to answer the question of whether cancer-related anxiety and depression are modulated in the same way as the psychiatric findings imply. Mindfulness exercises also show an increase of alpha band and are already used for the treatment of cancer patients. Like other authors,^4^ we see the need for studies that compare NF to other clinical approaches that promote healthy brain self-regulation, such as physical activity and mindfulness-based stress reduction strategies. In addition, more information is needed about which NF technologies, approaches, and protocols could be successfully used with cancer patients or cancer survivors. It is important to recognize the strength of non-qEEG based systems to implement less-expensive interventions in psycho-oncology and the use of replicable (eg, fixed sensor placement) and patient-friendly protocols. Clinical trials comparing the effectiveness of specific NF protocols and approaches to sham neurofeedback and/or other NF protocols are urgently needed to move this field forward. Study limitations should be addressed in future research by using randomization and double-blinding, larger sample sizes to increase the quality of individual studies, as well as uniform, international, and standardized measurement instruments.

This review shows that further NF research in the field of cancer therapy is needed to assess the efficacy and to uncover the underlying mechanisms. In the long term, the goal should be to alleviate the usual symptoms of this patient population and increase the quality of life. The results will be important for noninvasive, drug-free symptom management in cancer survivors.

## References

[1] Micoulaud-Franchi JA McGonigal A Lopez R Daudet C Kotwas I Bartolomei F. Electroencephalographic neurofeedback: level of evidence in mental and brain disorders and suggestions for good clinical practice. Neurophysiol Clin. 2015;45:423-433. doi:10.1016/j.neucli.2015.10.077 26553293

[2] Kamiya J. Biofeedback training in voluntary control of EEG alpha rhythms. Calif Med. 1971;115:44. PMC1518077 18730594

[3] Milstein V. Contingent alpha blocking: conditioning or sensitization? Electroencephalogr Clin Neurophysiol. 1965;18:272-277. doi:10.1016/0013-4694(65)90093-3 14255055

[4] Luctkar-Flude M Groll D. A systematic review of the safety and effect of neurofeedback on fatigue and cognition. Integr Cancer Ther. 2015;14:318-340. doi:10.1177/1534735415572886 25716351

[5] Collura TF Technical Foundations of Neurofeedback. 1st ed. (Routledge Monograph Series on Neurotherapy and QEEG Neuroscience Techniques). New York, NY: Routledge; 2014.

[6] Glombiewski JA Bernardy K Häuser W. Efficacy of EMG- and EEG-biofeedback in fibromyalgia syndrome: a meta-analysis and a systematic review of randomized controlled trials. Evid Based Complement Alternat Med. 2013;2013:962741. doi:10.1155/2013/962741 24082911 PMC3776543

[7] Teufel M Stephan K Kowalski A et al Impact of biofeedback on self-efficacy and stress reduction in obesity: a randomized controlled pilot study. Appl Psychophysiol Biofeedback. 2013;38:177-184. doi:10.1007/s10484-013-9223-8 23760668

[8] Othmer S Othmer S. Infra-low-frequency neurofeedback for optimum performance. Biofeedback. 2016;44:81-89. doi:10.5298/1081-5937-44.2.07

[9] Gannon L Sternbach RA. Alpha enhancement as a treatment for pain: a case study. J Behav Ther Exp Psychiatry. 1971;2:209-213. doi:10.1016/0005-7916(71)90061-9

[10] Caro XJ Winter EF. EEG biofeedback treatment improves certain attention and somatic symptoms in fibromyalgia: a pilot study. Appl Psychophysiol Biofeedback. 2011;36:193-200. doi:10.1007/s10484-011-9159-9 21656150

[11] Kayiran S Dursun E Ermutlu N Dursun N Karamürsel S. Neurofeedback in fibromyalgia syndrome. Agri. 2007;19:47-53. 18095199

[12] Sime A. Case study of trigeminal neuralgia using neurofeedback and peripheral biofeedback. J Neurother. 2004;8:59-71. doi:10.1300/J184v08n01_05

[13] Jensen MP Grierson C Tracy-Smith V Bacigalupi SC Othmer S. Neurofeedback treatment for pain associated with complex regional pain syndrome type I. J Neurother. 2007;11:45-53. doi:10.1300/J184v11n01_04

[14] Windthorst P Mazurak N Kuske M et al Heart rate variability biofeedback therapy and graded exercise training in management of chronic fatigue syndrome: an exploratory pilot study. J Psychosom Res. 2017;93:6-13. doi:10.1016/j.jpsychores.2016.11.014 28107894

[15] Schoenberg PLA David AS. Biofeedback for psychiatric disorders: a systematic review. Appl Psychophysiol Biofeedback. 2014;39:109-135. doi:10.1007/s10484-014-9246-9 24806535

[16] Buzsáki G Logothetis N Singer W. Scaling brain size, keeping timing: evolutionary preservation of brain rhythms. Neuron. 2013;80:751-764. doi:10.1016/j.neuron.2013.10.002 24183025 PMC4009705

[17] Herrmann CS Knight RT. Mechanisms of human attention: event-related potentials and oscillations. Neurosci Biobehav Rev. 2001;25:465-476. 11595268 10.1016/s0149-7634(01)00027-6

[18] Hegerl U Hensch T. The vigilance regulation model of affective disorders and ADHD. Neurosci Biobehav Rev. 2014;44:45-57. doi:10.1016/j.neubiorev.2012.10.008 23092655

[19] Johnston SJ Boehm SG Healy D Goebel R Linden DEJ Neurofeedback: a promising tool for the self-regulation of emotion networks. Neuroimage. 2010;49:1066-1072. doi:10.1016/j.neuroimage.2009.07.056 19646532

[20] Halson SL. Neurofeedback as a potential nonpharmacological treatment for insomnia. Biofeedback. 2017;45:19-20. doi:10.5298/1081-5937-45.1.08

[21] Oken BS Salinsky MC Elsas SM. Vigilance, alertness, or sustained attention: Physiological basis and measurement. Clin Neurophysiol. 2006;117:1885-1901. doi:10.1016/j.clinph.2006.01.017 16581292 PMC2865224

[22] Haenschel C Baldeweg T Croft RJ Whittington M Gruzelier J. Gamma and beta frequency oscillations in response to novel auditory stimuli: a comparison of human electroencephalogram (EEG) data with in vitro models. Proc Natl Acad Sci U S A. 2000;97:7645-7650. doi:10.1073/pnas.120162397 10852953 PMC16599

[23] Strijkstra AM Beersma DGM Drayer B Halbesma N Daan S. Subjective sleepiness correlates negatively with global alpha (8-12 Hz) and positively with central frontal theta (4-8 Hz) frequencies in the human resting awake electroencephalogram. Neurosci Lett. 2003;340:17-20. 12648748 10.1016/s0304-3940(03)00033-8

[24] Niedermeyer E. Alpha rhythms as physiological and abnormal phenomena. Int J Psychophysiol. 1997;26:31-49. 9202993 10.1016/s0167-8760(97)00754-x

[25] Davidson RJ. Anterior electrophysiological asymmetries, emotion, and depression: conceptual and methodological conundrums. Psychophysiology. 1998;35:607-614. 9715104 10.1017/s0048577298000134

[26] Stewart JL Bismark AW Towers DN Coan JA Allen JJB Resting frontal EEG asymmetry as an endophenotype for depression risk: sex-specific patterns of frontal brain asymmetry. J Abnorm Psychol. 2010;119:502-512. doi:10.1037/a0019196 20677839 PMC2916182

[27] American Psychiatric Association. Diagnostic and Statistical Manual of Mental Disorders. 5th ed. Washington, DC: American Psychiatric Association; 2013.

[28] Davidson RJ Pizzagalli D Nitschke JB Putnam K. Depression: perspectives from affective neuroscience. Annu Rev Psychol. 2002;53:545-574. doi:10.1146/annurev.psych.53.100901.135148 11752496

[29] Torre LA Bray F Siegel RL Ferlay J Lortet-Tieulent J Jemal A. Global cancer statistics, 2012. CA Cancer J Clin. 2015;65:87-108. doi:10.3322/caac.21262 25651787

[30] Siegel R Naishadham D Jemal A. Cancer statistics, 2013. CA Cancer J Clin. 2013;63:11-30. doi:10.3322/caac.21166 23335087

[31] Ferlay J Steliarova-Foucher E Lortet-Tieulent J et al Cancer incidence and mortality patterns in Europe: estimates for 40 countries in 2012. Eur J Cancer. 2013;49:1374-1403. doi:10.1016/j.ejca.2012.12.027 23485231

[32] Jemal A Vineis P Bray F Torre L Forman D. The Cancer Atlas. 2nd ed. Atlanta, GA: American Cancer Society; 2014.

[33] de Moor JS Mariotto AB Parry C et al Cancer survivors in the United States: Prevalence across the survivorship trajectory and implications for care. Cancer Epidemiol Biomarkers Prev. 2013;22:561-570. doi:10.1158/1055-9965.EPI-12-1356 23535024 PMC3654837

[34] Falquez R Couto B Ibanez A et al Detaching from the negative by reappraisal: the role of right superior frontal gyrus (BA9/32). Front Behav Neurosci. 2014;8:165. doi:10.3389/fnbeh.2014.00165 24847230 PMC4023069

[35] Prinsloo S Gabel S Lyle R Cohen L. Neuromodulation of cancer pain. Integr Cancer Ther. 2014;13:30-37. doi:10.1177/1534735413477193 23439659

[36] Moher D Liberati A Tetzlaff J Altman DG; PRISMA Group. Preferred reporting items for systematic reviews and meta-analyses: the PRISMA statement. Int J Surg. 2010;8:336-341. doi:10.1016/j.ijsu.2010.02.007 20171303

[37] Liberati A Altman DG Tetzlaff J et al The PRISMA statement for reporting systematic reviews and meta-analyses of studies that evaluate health care interventions: explanation and elaboration. PLoS Med. 2009;6:e1000100. doi:10.1371/journal.pmed.1000100 19621070 PMC2707010

[38] Alvarez J Meyer FL Granoff DL Lundy A. The effect of EEG biofeedback on reducing postcancer cognitive impairment. Integr Cancer Ther. 2013;12:475-487. doi:10.1177/1534735413477192 23584550

[39] Nelson DV Esty ML. Neurotherapy as a catalyst in the treatment of fatigue in breast cancer survivorship. Explore (NY). 2016;12:246-249. doi:10.1016/j.explore.2016.04.002 27234465

[40] Prinsloo S Novy D Driver L et al Randomized controlled trial of neurofeedback on chemotherapy-induced peripheral neuropathy: a pilot study. Cancer. 2017;123:1989-1997. doi:10.1002/cncr.30649 28257146 PMC5470539

[41] Prinsloo S Novy D Driver L et al The long-term impact of neurofeedback on symptom burden and interference in patients with chronic chemotherapy-induced neuropathy: analysis of a randomized controlled trial. J Pain Symptom Manage. 2018;55:1276-1285. doi:10.1016/j.jpainsymman.2018.01.010 29421164

[42] Prinsloo S Gabel S Park L Lyle R. Neurofeedback for chemotherapy induced neuropathic symptoms: a case study. NeuroRegulation. 2014;1:165-172. doi:10.15540/nr.1.2.165

[43] de Ruiter MA Oosterlaan J Schouten-van Meeteren AYN et al Neurofeedback ineffective in paediatric brain tumour survivors: results of a double-blind randomised placebo-controlled trial. Eur J Cancer. 2016;64:62-73. doi:10.1016/j.ejca.2016.04.020 27343714

[44] Windthorst P Veit R Enck P Smolka R Zipfel S Teufel M. Biofeedback und neurofeedback: applications in psychosomatic medicine and psychotherapy [in German]. Psychother Psychosom Med Psychol. 2015;65:146-158. doi:10.1055/s-0034-1387320 25798668

[45] Gorini A Marzorati C Casiraghi M Spaggiari L Pravettoni G. A neurofeedback-based intervention to reduce post-operative pain in lung cancer patients: study protocol for a randomized controlled trial. JMIR Res Protoc. 2015;4:e52. doi:10.2196/resprot.4251 PMC4436521 25940965

[46] Chiesa A Serretti A. Mindfulness-based stress reduction for stress management in healthy people: a review and meta-analysis. J Altern Complement Med. 2009;15:593-600. doi:10.1089/acm.2008.0495 19432513

